# A randomized comparison of loss of resistance versus loss of resistance plus electrical stimulation: effect on success of thoracic epidural placement

**DOI:** 10.1186/s12871-022-01584-x

**Published:** 2022-02-09

**Authors:** Sean Wayne Dobson, Robert Stephen Weller, Christopher Edwards, James David Turner, Jonathan Douglas Jaffe, Jon Wellington Reynolds, Daryl Steven Henshaw

**Affiliations:** Department of Anesthesiology, Wake Forest University School of Medicine, 9th Floor Janeway Tower One Medical Center Boulevard, Winston Salem, NC 27157 USA

**Keywords:** Acute pain management, Analgesia, Loss of resistance, Nerve stimulation, Regional anesthesia, Thoracic epidural

## Abstract

**Background:**

Loss of resistance (LOR) for epidural catheter placement has been utilized for almost a century. LOR is a subjective endpoint associated with a high failure rate. Nerve stimulation (NS) has been described as an objective method for confirming placement of an epidural catheter. We hypothesized that the addition of NS to LOR would improve the success of epidural catheter placement.

**Methods:**

One-hundred patients were randomized to thoracic epidural analgesia (TEA) utilizing LOR-alone or loss of resistance plus nerve stimulation (LOR + NS). The primary endpoint was rate of success, defined as loss of sensation following test dose. Secondary endpoints included performance time. An intention-to-treat analysis was planned, but a per-protocol analysis was performed to investigate the success rate when stimulation was achieved.

**Results:**

In the intention-to-treat analysis there was no difference in success rates (90% vs 82% [LOR + NS vs LOR-alone]; *P* = 0.39). The procedural time increased in the LOR + NS group (33.9 ± 12.8 vs 24.0 ± 8.0 min; *P* < 0.001). The per-protocol analysis found a statistically higher success rate for the LOR + NS group compared to the LOR-alone group (98% vs. 82%; *P* = 0.017) when only patients in whom stimulation was achieved were included.

**Conclusions:**

Addition of NS technique did not statistically improve the success rate for epidural placement when analyzed in an intention-to-treat format and was associated with a longer procedural time. In a per-protocol analysis a statistically higher success rate for patients in whom stimulation was obtained highlights the potential benefit of adding NS to LOR.

**Trial registration:**

ClinicalTrials.gov identifier NCT03087604 on 3/22/2017; Institutional Review Board Wake Forest School of Medicine IRB00039522, Food and Drug Administration Investigational Device Exemption: G160273.

**Supplementary Information:**

The online version contains supplementary material available at 10.1186/s12871-022-01584-x.

## Background

Loss of resistance (LOR) for identifying the epidural space, first described in 1933, is still used today [1]. In this technique a provider engages the *ligamentum flavum* with a needle, which provides resistance to injection. The needle is advanced with serial attempts at injection until resistance is “lost,” suggesting that the needle has advanced through the ligament into the epidural space.

Catheters placed utilizing LOR have a reported failure rate between 15–30% [2–8]. A patient’s habitus, spinal anatomy, level of experience, and efficiency pressures may all impact success. The endpoint in the LOR technique is subjective and determined solely by the person performing the procedure. The LOR lacks specificity as “false losses” can occur when the needle enters a potential space. Resistance to threading a catheter may suggest that the needle is improperly located, but the feel during catheter advancement after “false-losses” can mimic threading into the epidural space.

Confirmation of catheter placement following LOR involves checking for a loss of sensation to cold following a test dose of local anesthetic. When sensory changes do not occur, the catheter is assumed to be outside the epidural space. Typically, during this time sterile dressings are placed, drapes removed, and sterility broken. Frequently, patients are also moved to the operating room and/or placed under general anesthesia making it less likely that improperly placed catheters are recognized. Even in the event that recognition occurs, replacement requires an additional procedure with associated additional risks, supplies, and time.

Given the limitations of LOR, alternative approaches for identifying the epidural space and confirming proper catheter placement have been described. One such approach involves nerve stimulation (NS). In this technique electrical current is passed through the catheter tip in an attempt to evoke muscle contractions in the abdomen or chest wall through stimulation of spinal nerve roots. This provides an objective endpoint, does not require patient participation, and does not require waiting for the onset of a pharmacologic agent or disturbance of the sterile field, which allows the procedure to be immediately repeated if NS is not achieved.

Although NS is relatively simple to perform and was first described in 1998, it has not gained widespread use [9]. The benefit of adding NS to the traditional LOR has not been adequately explored. This study was designed to compare the LOR-alone vs. LOR + NS under the hypothesis that NS would improve the success rate of catheter placement for thoracic analgesia.

## Methods

The study was designed as a prospective, observer-blinded, randomized, traditional comparative study with a target enrollment of 100 patients between March and October of 2017 with 50 in each group.

### Ethics approval and study registration

This trial was registered at ClinicalTrials.gov (identifier NCT03087604 on 3/22/2017). Institutional review board approval from Wake Forest University Health Sciences was obtained (IRB00039522) before the start of this study. A US Food and Drug Administration (FDA) Investigational Device Exemption (IDE) was also obtained to allow for the off-label use of the StimuCath® Continuous Nerve Block Catheter (Arrow by Teleflex Medical, Morrisville, NC USA) in the epidural space and for subsequent NS (FDA:G160273). Informed consent was obtained from all participants. All methods were performed in accordance within the relevant guidelines and regulations of the institutional review board and the food and drug administration investigational device exemption.

### Consent to participate

Not applicable.

### Availability of data and materials

All data is contained in the supplemental table of this manuscript and is also publicly available at https://clinicaltrials.gov/ct2/show/NCT03087604?term=dobson+epidural&draw=2&rank=1.

### Inclusion criteria

Patients between the ages of 18 and 90 who were undergoing thoracic or abdominal surgery and whose surgeon requested postoperative epidural analgesia were eligible for the study.

### Exclusion criteria

Exclusion criteria included an allergy to amide local anesthetics, presence of a progressive neurological deficit, coagulopathy, or recent anticoagulant administration inconsistent with American Society of Regional Anesthesia and Pain Medicine (ASRA) guidelines [10] for timing of neuraxial block, systemic infection or infection at the site of planned placement, and patient refusal or the inability to give informed consent.

### Methodology

All epidurals were placed in a dedicated regional procedural holding area outside of the operating room. After informed consent was obtained, an intravenous (IV) catheter was placed, standard American Society of Anesthesiologists (ASA) monitors and oxygen by facemask was applied. Patients were positioned lateral decubitus and premedicated with IV midazolam and fentanyl at the discretion of the supervising attending anesthesiologist. Randomization by a random number generator was performed and the group randomization was placed in a sealed envelope by a third party. After consent, sequential envelopes were selected and opened for group randomization by the person obtaining consent.

All epidurals were performed by a senior resident (clinical anesthesia year 2 or clinical anesthesia year 3) or a regional anesthesia and acute pain management fellow who was supervised by an attending anesthesiologist who regularly performs epidural procedures. The attending anesthesiologist had the option of taking over the epidural procedure in either group if three unsuccessful attempts were made or a time limit of thirty minutes was exhausted. To assist with observer blinding, all patients received a 19 Ga × 90 cm StimuCath® Continuous Nerve Block Catheter with SnapLock™ Adapter (Arrow by Teleflex Medical, Morrisville, NC USA). The spinal level of epidural placement was determined by the attending anesthesiologist as appropriate for the surgery and a paramedian approach in the lateral position was consistently utilized for all epidurals. The Tuohy needles were inserted 1 cm lateral to the end of the spinous process and then advanced perpendicular in all planes until contact was made with the lamina. The needle was walked off the lamina at an angle of 45 degrees rostrally and 20 degrees medially until the edge of the lamina was detected. The needle was then advanced over the edge of the lamina until the engagement of the ligamentum flavum was detected. The needle was then advanced through the ligamentum flavum until a loss of resistance to 2 ml of air was achieved on the plunger of a LOR syringe. All epidurals were placed using air for LOR.

In the traditional LOR technique group catheters were threaded following convincing LOR determined by the physician performing the procedure. If resistance was met during threading of the catheter the provider was free to re-approach the epidural space and/or change spinal levels as many times as necessary until they were satisfied with the LOR, and the catheter threaded easily.

In the LOR + NS group, epidural catheters were placed using traditional LOR followed by the use of NS to evaluate for truncal muscle contractions. In this group catheters were also threaded following LOR. Again, if resistance was met during threading of the catheter the provider was free to re-approach the epidural space and/or change spinal levels as needed until they were satisfied with the LOR and the catheter threaded easily. Once the catheter was placed the sterile Tuohy Borst connector was attached and electrical current was applied using a peripheral nerve stimulator (B Braun, HNS12, Bethlehem, PA USA). Initial stimulator settings included a stimulus frequency of 2 Hz, pulse duration of 0.3 ms, and current of 0.5 mA. The patient’s abdomen and chest were inspected and/or palpated to determine if muscle contractions were present. If none were appreciated the current was slowly increased to 5.0 mA (the maximal output on the stimulator). If contractions were still not present the pulse duration was increased first to 0.5 ms and then to 1.0 ms with the current being increased slowly at each setting from 0.5 mA to 5 mA based on Ban Tsui’s work using NS up to 10 mA to confirm epidural catheter placement where criteria for successful catheter placement was established [9]. If appropriate myotomal stimulation was achieved at any point the procedure was considered to be complete. If no motor stimulation was present at 1.0 ms and 5 mA, the catheter and needle were withdrawn and the provider was free to reattempt the procedure until convincing LOR and positive muscle stimulation was elicited. If attempts at multiple levels were required, and/or OR readiness mandated completion of the procedure (soft cut-off of 30 min) the supervising attending anesthesiologist had the discretion to accept the LOR and catheter threading in the absence of stimulation in the LOR + NS group so as to avoid delaying the surgical case.

After catheter placement in both groups a test dose was performed using 3 ml of 1.5% lidocaine with 1:200,000 (5 mcg/ml) epinephrine. After appropriately ensuring that the catheter was neither intravenous (by looking for a change in heart rate or systolic blood pressure) nor subarachnoid (by checking for the ability to move the lower extremities), an additional 2 ml was administered. Fifteen minutes after the full lidocaine dose, a member of the study team blinded to group assignment tested for procedural success by evaluating for a loss of cold sensation to ice. After assessment a sterile bandage was applied to the catheter site. Successful catheter placement was defined as a loss of cold sensation in at least two bilateral contiguous dermatomes in the anterior thorax or abdomen [8, 9]. If a loss of cold sensation did not occur the epidural catheter placement was classified as unsuccessful.

In both groups, a procedural time limit of 30 min was recommended, but faculty could elect to take over and continue the procedure if deemed in the best interest of the patient, so some procedures exceeded the 30-min mark. All patients received general anesthesia for their surgical case.

The primary outcome of the study was the success rates for the two approaches to epidural catheter placement. Secondary outcomes included procedural performance time recorded in minutes from the time of the pre-procedural timeout until the test dose was administered.

### Statistical analysis

On the basis of a literature search for the expected failure rate for thoracic epidural catheter placement at academic institutions with conventional LOR technique, we expected a 23% failure rate (success rate of 77%) [8]. We hypothesized that the use of NS would decrease the failure rate to 3% (success rate of 97%) [8]. A sample size of 44 patients per group was calculated as being required for a statistical power of 0.8 and a type 1 error of 0.05 using a Chi squared test. To allow for potential dropout due to inability to place a catheter, a total of 100 subjects were targeted for the study. Patients were evaluated in an intention-to-treat model (a priori), but a post-hoc, per protocol analysis was also performed to further define the potential utility of nerve stimulation. This was performed due to the inability to achieve stimulation in a few of the patients in the LOR + NS group due to pre-surgical time constraints. In the post hoc per-protocol analysis, patients not obtaining stimulation were dropped from the stimulation group due to non-completion of the protocol. Two-tailed Fisher’s exact test was used to assess statistical significance between the LOR and the LOR + NS groups. Using the observed proportions in each group, the difference in the rates was estimated by calculating the standard deviation of the sampling distribution and using that information to calculate a 95% confidence interval around the difference using the Wald formula. SAS version 9.4 (Cary, NC USA).

## Results

**Fig. 1:**
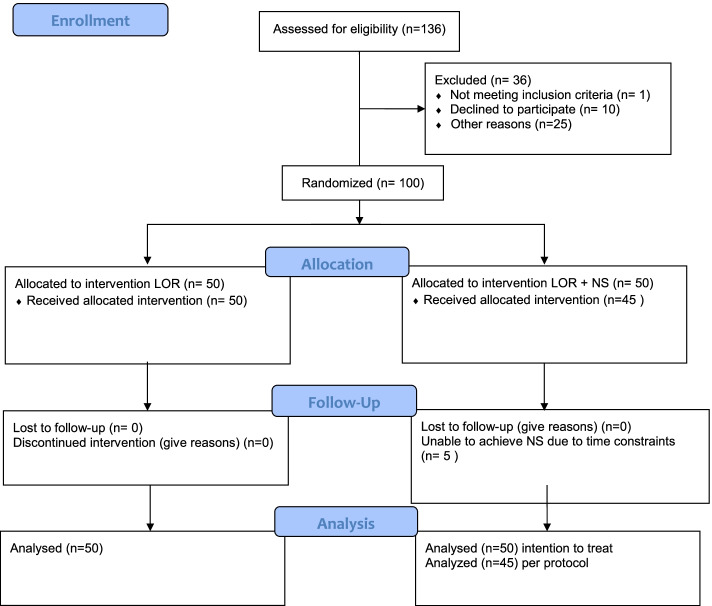
Consort diagram of the study

One hundred total patients, 50 per group, were recruited over a period of seven months from March of 2017 until October of 2017. The consort diagram is depicted in Fig. 1. Demographic data for the study participants are shown in Table 1.

**Table 1:** Patient characeteristics

	LOR	NS-LOR
Mean Age (yr)	63.2 ± 12.6	60.4 ± 15.4
Sex (M/F)	31/19	23/27
Mean Body mass index (kg/m^2^)	27.9 ± 5.8	29.0 ± 6.12
Ethnicity (Caucasian/African American/Asian/ Hispanic)	45/3/1/1	46/4/0/0
Site of surgery (Thoracic/Abdominal)	12/38	15/35
ASA Status (I/II/III/IV)	0/2/48/0	0/3/46/1

No significant differences

*LOR* loss of resistance, *NS* nerve stimulation, *ASA* American Society of Anesthesiologist

There were 44 catheters in the LOR + NS group which achieved stimulation and were considered successful. One catheter achieved stimulation, but was deemed a failure as no loss of sensation occurred. An additional single catheter did not achieve stimulation but did result in a loss of cold sensation and was considered successful. The four remaining catheters that did not achieve stimulation also did not result in a loss of cold sensation and were considered failures. There were 41 catheters in the LOR-alone group that were deemed successful while 9 catheters did not result in a loss of cold sensation (Table 2).

**Table 2:** Success of epidural catheter placement

	Successful	Failed
Intention to Treat		
LOR + NS		
NS Achieved	**44**	**1**
NS not achieved	**1**	**4**
LOR		
LOR achieved	**41**	**9**
**Per Protocol**		
LOR + NS		
NS Achieved	**44**	**1**
LOR		
LOR achieved	**41**	**9**

*LOR* Loss of resistance, *NS* nerve stimulation

Based on the intention-to-treat analysis, the overall success rate for catheters placed using LOR + NS was 90% (45/50) while the overall success rate for catheters placed using LOR-alone was 82% (41/50); (*P* = 0.39; estimated difference of 8% (95% CI = –5.6, to + 21.6%).

The secondary outcome of procedural time was found to be significantly longer in the LOR + NS group as compared to the LOR-alone (33.9 ± 12.8 min vs. 24.0 ± 8.0; *P* =  < 0.008).

The per-protocol analysis, which included only those patients in the LOS + NS group in whom stimulation was ultimately achieved, resulted in success rates of 98% (44/45) in the LOR + NS group and 82% (41/50) in the LOR-alone group. A comparison of these success rates was statistically different; (*P* = 0.017; estimated difference of 16% (95% CI = 4.3% to 27.3%).

## Discussion

This study aimed to assess the value of adding NS to LOR for placement of thoracic epidurals. Although the success rate within the LOR + NS group was higher compared to LOR-alone, our a priori intention-to-treat analysis failed to show a statistical benefit in terms of success. The authors acknowledge that the impact that time-pressures had on the ability to achieve the endpoint of NS in a few of the patients in the LOR + NS group was likely underestimated. The protocol was designed to allow the anesthesiologist to accept LOR after attempts at multiple levels in the LOR + NS group if procedural time was prolonged so as not delay the surgery. Unfortunately, this resulted in five LOR + NS subjects having their catheter placement accepted after only LOR.

In hindsight, the protocol could have allowed for a longer time, or specified exclusion, if NS was not achieved. Ethically, because an intention-to-treat analysis was originally planned, data were presented in that format and the conclusions of the study are based solely on that analysis. However, such results could misrepresent the value of NS since five patients never achieved NS and were essentially identical to LOR. Therefore, we performed the per-protocol analysis where these five subjects were essentially excluded as the lack NS technically represented a protocol deviation. In this analysis the success rate in the LOR + NS group was statistically higher compared to LOR alone.

In both analyses the success rate of LOR alone for epidural catheter placement (82%) was relatively consistent with what was expected in the power analysis (77%) and those previously published (20–25%) [2, 8, 11]. However, the improved success rate that was obtained with LOR and the five subjects that did not obtain stimulation resulted in the intention to treat analysis not being statistically significant despite an eight percent improvement in successful epidural catheter placement.

One concern of NS is that procedural time may be longer as more steps are involved. While this concern is validated by our study, it is not surprising. The first time that a LOR occurred in the LOR-alone group a test dose was administered. However, if a LOR occurred in the LOR + NS group, but NS was not obtained, the procedure was repeated until NS was obtained or the soft time-limit was reached. Therefore, it is expected that longer times would occur in the LOR + NS group, especially since the high failure rate in the LOR group suggests that it is unlikely that NS would occur consistently after the first LOR. The important benefits of a higher success rate with NS likely warrants the additional time required. These results likely simulate real-world clinical situations with respect to time.

In the last few years, a number of approaches for confirming epidural catheter placement, ranging from epidurograms, the use of ultrasound to identify landmarks, to pressure waveform analysis, have been published [8]. The use of fluoroscopy with epidurogram has been demonstrated to be a highly effective method of determining the correct placement of an epidural catheter [12]. Despite the efficacy of fluoroscopy, the use of fluoroscopy has not become widespread due to radiation exposure, availability, and the added cost and time. The use of pressure waveform analysis has been shown to improve the success rate of epidural catheter placement as compared to LOR alone [8]. Pressure waveform analysis is inexpensive, and the tools needed to perform pressure wave analysis are readily available, but waveform analysis can be difficult to interpret. The use of ultrasound has been proposed to assist in the placement of a thoracic epidural catheter but no randomized controlled trials evaluating the utility of ultrasound have been published [13, 14]. While these techniques may have utility, studies comparing them to NS and to each other are needed. The advantage of NS is that it does not expose patients to contrast or radiation and it can be conducted using equipment that is typically already available to anesthesiologists. Despite being described over 20 years ago [9, 15–18], its use has not been widely adopted. This may be due to the perceived complexity of setting up a circuit with conventional catheters and a saline bridge [18]. In this study, we demonstrate that the use of a peripheral stimulating nerve catheter can simplify the process of NS, but this was an off-label use of this device—an FDA IDE was obtained for this study. However, this does not preclude NS use and the authors have utilized this technique over the past 5 years in approximately 2000 patients without any perceived side-effects, such as increased rates of infection, dural puncture, intravenous catheters, or epidural hematoma, and significantly decreased our failed epidural catheter placements. Perhaps, if NS gains a foothold then stimulating catheters approved specifically for use in the epidural space may become available.

Clinically, the results of this study are highly relevant and are used daily by the authors to guide clinical decision making. If NS is achieved following LOR, clinicians can have a high level of confidence in the post-procedure functionality of the epidural. However, if LOR occurs, but NS is not present, it is highly likely, but not absolute, that the epidural catheter has been placed outside the epidural space.

This study has several limitations worth considering. First, the original power analysis was based on a previously published study in which a Pearson’s Chi-squared test was used to calculate sample size; resulting in the calculation of 44 patients needed per group. However, since the Pearson’s Chi-squared test may not be entirely accurate when the expected cell count is < 5 and the sample size is < 100, it may be suggested that a Fisher’s exact test would be a more prudent choice. While likely true, it should be noted that a power analysis utilizing the Fisher’s exact test would have calculated a sample size of 49 patients per group, which this study ultimately exceeded given the complete lack of patient drop-out or data loss.

Second, all procedures were initially performed by physicians in training. Not only could this have impacted both success rates and performance times, it may also suggest that the results of this study are less applicable to a clinical practice without learners. However, it should be noted that each procedure was closely supervised by an attending anesthesiologist with either fellowship training in regional anesthesia or significant experience in the specialty, which should theoretically decrease the potential impact of this “limitation.” In addition, for any other training institution the design of this study should closely replicate everyday clinical practice. Lastly, it was not possible to entirely blind the patient, which introduces the potential risk of bias. It is conceivable that the presence of muscle contractions alerted the patient to their group assignment, but we felt that the level of sedation reduced this chance. While the clinician placing the epidural was not blinded, all post-procedural assessments were performed by a blinded observer.

## Conclusions

In conclusion, the combination of NS and LOR did not statistically improve the success rate for thoracic epidural catheter placement compared to LOR alone when analyzed in an intention-to-treat format and was associated with a longer procedural time. Although not statistically significant, the 8% improvement in success could be considered a clinically significant difference to both providers and patients. In addition, when stimulation was actually achieved, which occurred in the vast majority of patients, the resultant success rate in a per-protocol analysis was statistically greater, which further highlights the potential benefit of NS for epidural catheter placement and calls for more research around this modality.

## Supplementary Information

**Additional file 1.**

## Data Availability

All raw data is included in the supplemental table of this manuscript as well as publicly available at: https://clinicaltrials.gov.

## Abbreviations

LOR: Loss of resistance
TEA: Thoracic epidural analgesia
NS: Nerve Stimulation
LOR + NS: Loss of resistance with the addition of nerve stimulation
FDA: Food and drug administration
ASRA: American Society of regional Anesthesia
IV: Intravenous
IDE: Investigational device exemption
Hz: Hertz
mA: Milliampere
