# Supplementary figures and images for: A randomized comparison of loss of resistance versus loss of resistance plus electrical stimulation: effect on success of thoracic epidural placement
Source: BMC Anesthesiol. 2022 Feb 9;22:43. doi: 10.1186/s12871-022-01584-x (PMC8826655; doi:10.1186/s12871-022-01584-x)

**
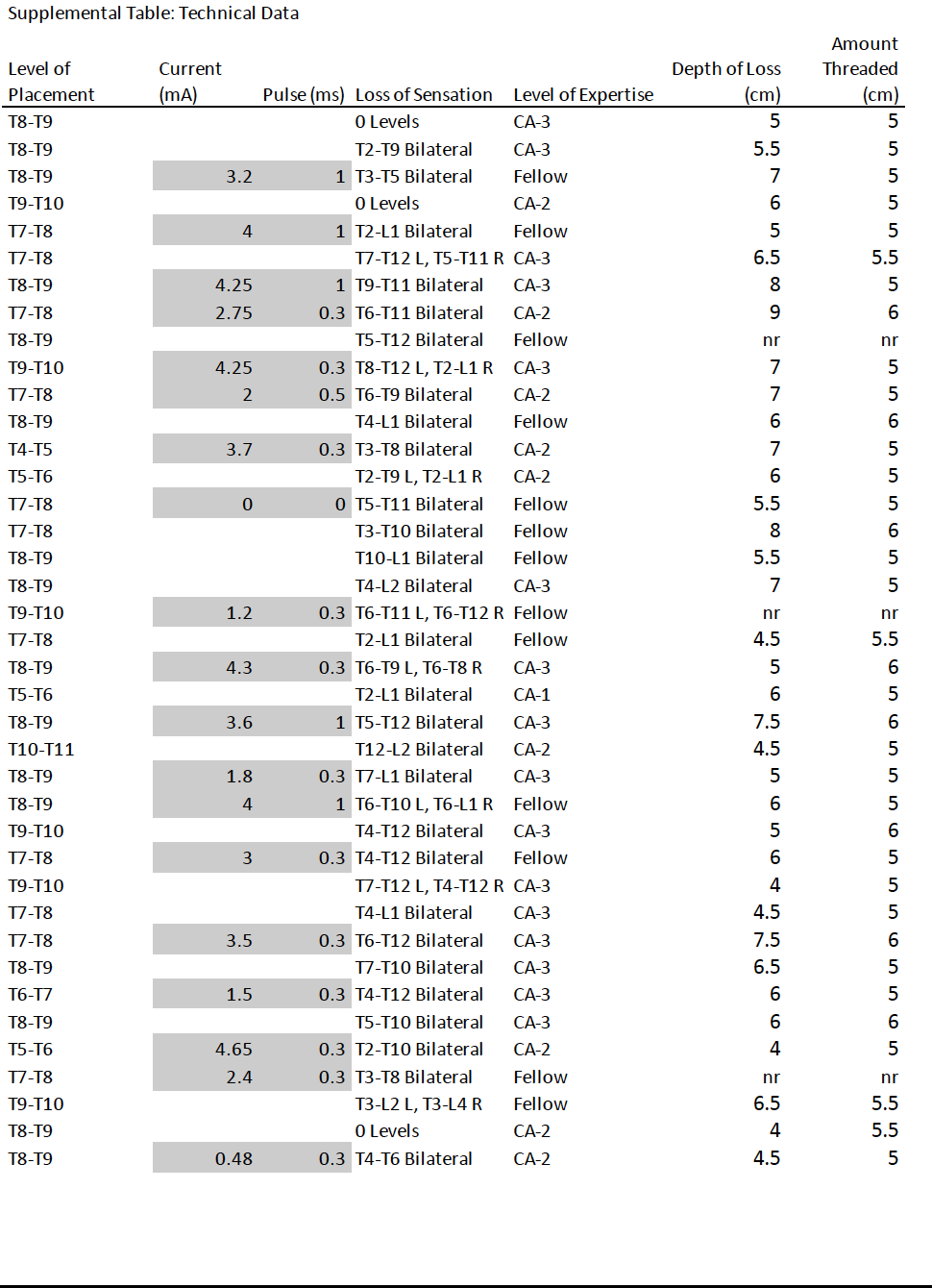
**

**
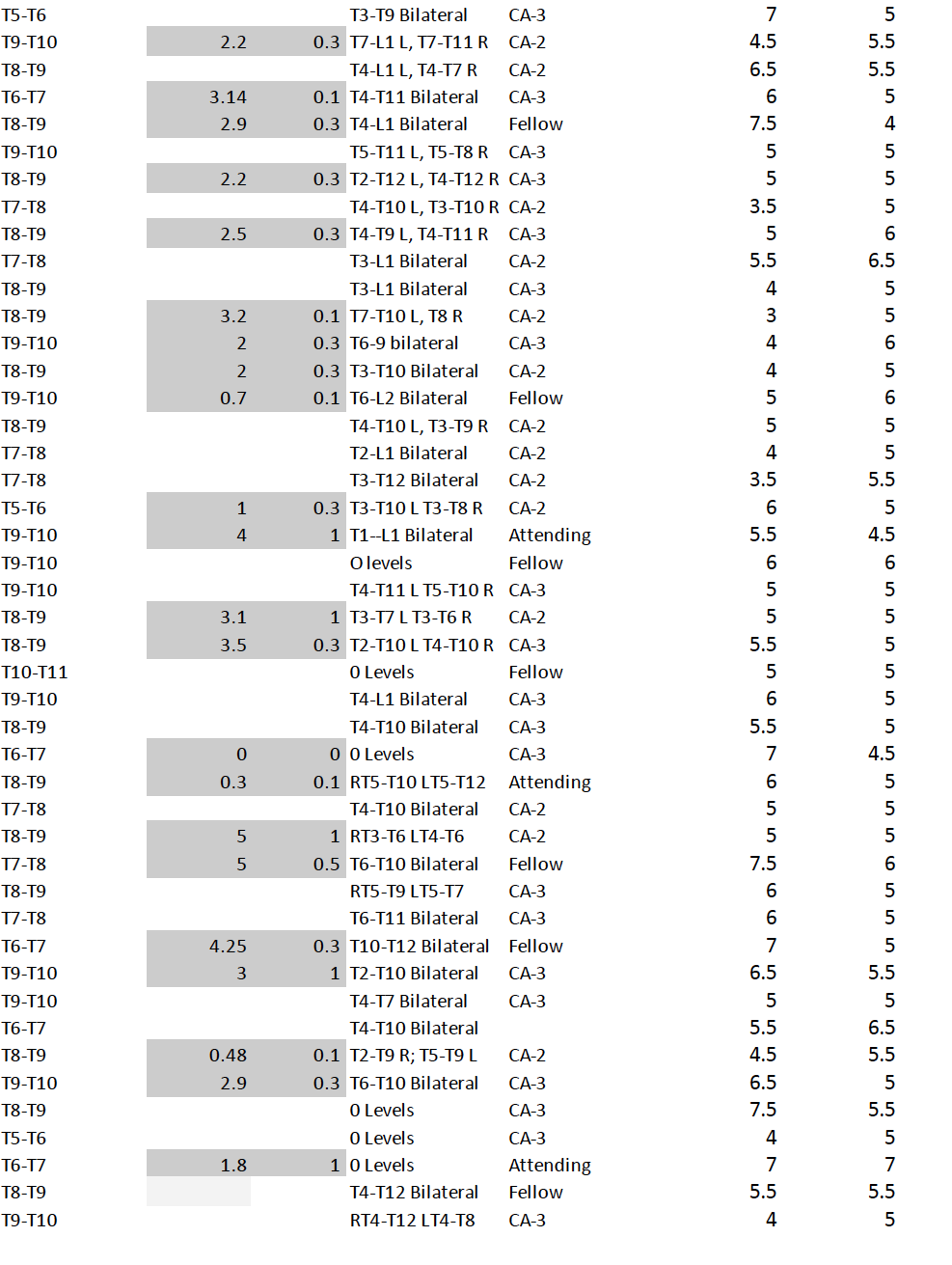
**

**
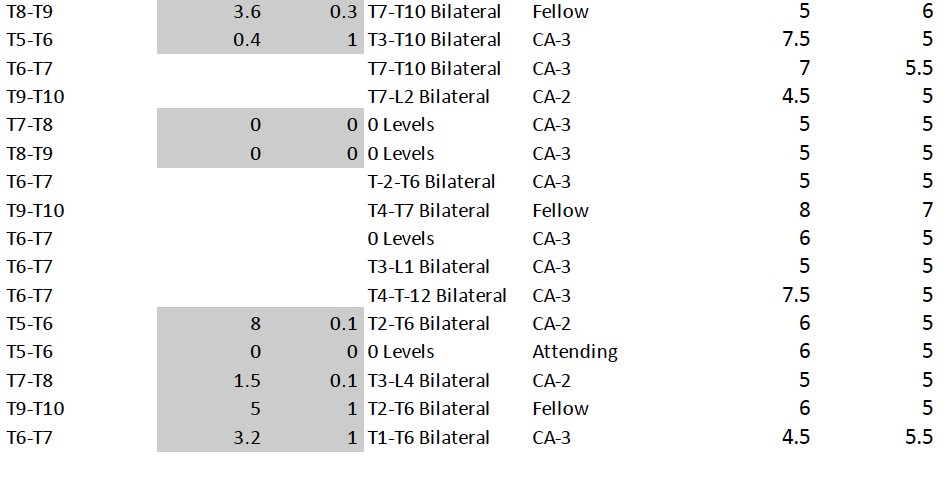
**

Supplement: Supplementary file 1 — Additional file 1. [file 12871_2022_1584_MOESM1_ESM.docx]
